# Inflammation and microbial translocation measured prior to combination antiretroviral therapy (cART) and long-term probability of clinical progression in people living with HIV

**DOI:** 10.1186/s12879-021-06260-y

**Published:** 2021-06-12

**Authors:** Esther Merlini, Alessandro Cozzi-lepri, Antonella Castagna, Andrea Costantini, Sergio Lo Caputo, Stefania Carrara, Eugenia Quiros Roldan, Maria A. Ursitti, Andrea Antinori, Antonella D’Arminio Monforte, Giulia Marchetti

**Affiliations:** 1 Clinic of Infectious Diseases, Department of Health Sciences, University of Milan, “ASST Santi Paolo e Carlo, Milan, Italy; 2 Institute for Global Health, University College London, London, UK; 3 Department of Infectious Diseases, IRCCS San Raffaele Scientific Institute, University Vita-Salute San Raffael, Milan, Italy; 4 Clinical Immunology Unit, Azienda Ospedaliero-Universitaria Ospedali Riuniti, Marche Polytechnic University, Ancona, Italy; 5 Infectious Diseases Unit, University of Foggia, Foggia, Italy; 6 Microbiology Biobank and Cell Factory Unit, National Institute for Infectious Diseases ‘Lazzaro Spallanzani’ IRCCS, Rome, Italy; 7 University Department of Infectious and Tropical Diseases, University of Brescia and ASST Spedali Civili, Brescia, Italy; 8 Department of Infectious Diseases, S. Maria Nuova IRCCS Hospital, Reggio Emilia, Italy; 9 HIV/AIDS Department, INMI, L. Spallanzani, IRCCS, Rome, Italy

**Keywords:** HIV-infection, Inflammation, Microbial translocation, Clinical progression

## Abstract

**Background:**

Despite the effectiveness of cART, people living with HIV still experience an increased risk of serious non-AIDS events, as compared to the HIV negative population. Whether pre-cART microbial translocation (MT) and systemic inflammation might predict morbidity/mortality during suppressive cART, independently of other known risk factors, is still unclear. Thus, we aimed to investigate the role of pre-cART inflammation and MT as predictors of clinical progression in HIV+ patients enrolled in the Icona Foundation Study Cohort.

**Methods:**

We included Icona patients with ≥2 vials of plasma stored within 6 months before cART initiation and at least one CD4 count after therapy available. Circulating biomarker: LPS, sCD14, EndoCab, hs-CRP. Kaplan-Meier curves and Cox regression models were used. We defined the endpoint of clinical progression as the occurrence of a new AIDS-defining condition, severe non-AIDS condition (SNAEs) or death whichever occurred first. Follow-up accrued from the data of starting cART and was censored at the time of last available clinical visit. Biomarkers were evaluated as both binary (above/below median) and continuous variables (logescale).

**Results:**

We studied 486 patients with 125 clinical events: 39 (31%) AIDS, 66 (53%) SNAEs and 20 (16%) deaths. Among the analyzed MT and pro-inflammatory markers, hs-CRP seemed to be the only biomarker retaining some association with the endpoint of clinical progression (i.e. AIDS/SNAEs/death) after adjustment for confounders, both when the study population was stratified according to the median of the distribution (1.51 mg/L) and when the study population was stratified according to the 33% percentiles of the distribution (low 0.0–1.1 mg/L; intermediate 1.2–5.3 mg/L; high > 5.3 mg/L). In particular, the higher the hs-CRP values, the higher the risk of clinical progression (*p* = 0.056 for median-based model; *p* = 0.002 for 33% percentile-based model).

**Conclusions:**

Our data carries evidence for an association between the risk of disease progression after cART initiation and circulating pre-cART hs-CRP levels but not with levels of MT. These results suggest that pre-therapy HIV-driven pro-inflammatory milieu might overweight MT and its downstream immune-activation.

**Supplementary Information:**

The online version contains supplementary material available at 10.1186/s12879-021-06260-y.

## Introduction

Despite the unquestioned success of cART in recovering CD4 T-cell count and in preventing disease progression [1–3], a few-year survival gap has been described in large cohorts of successfully cART-treated people living with HIV (PLWHIV) when compared to HIV-negative individuals [4–6], mainly ascribable to an excess risk of serious non-AIDS events (SNAEs) (as reviewed in [7]).

This is particularly true among patients who initiate cART at advanced disease stages and fail to achieve a significant CD4 recovery upon virologically-suppressive cART [8–12]. Gloomily, to date, the proportion of individuals who receive an HIV diagnosis during advanced infection is still relevant globally. Indeed, a recent large study recruiting patients from 55 countries, showed that up to 20–30% of patients starting therapy in 2015 in high-income countries are diagnosed with CD4 count < 350 cell/mmc [13]. Accordingly, within the Icona cohort, patients presenting with CD4 < 350/cmm at HIV diagnosis account for 43% (data unpublished).

Persistently heightened immune activation and inflammation on cART have been associated with both inefficient CD4 recovery [14–17] and the onset of several non-AIDS defining conditions upon cART initiation [18–22].

In untreated HIV infection, our group and others have shown that the markers of immune activation derived from the systemic translocation of bacterial byproducts through a damaged gut mucosal barrier, mainly lipopolysaccharide (LPS), are independent predictors of disease progression [23, 24]. Interestingly, persistently elevated levels of circulating LPS have been described in patients with inefficient CD4 gain on virologically-suppressive cART [25], in turn correlating to a persisting immune activation [26–30], leading to speculate that microbial translocation (MT) might affect immune reconstitution on cART.

Among PLWHIV on suppressive cART in North America, markers of innate immune activation and inflammation were much more strongly predictive of mortality than T-cell activation [29, 30], possibly due to the non-infectious nature of the co-morbidities (e.g., cardiovascular, non-AIDS cancer, etc.). In contrast, in resource-constrained settings, where infectious complications remain much more important causes of death [31, 32], T-cell activation and defects in adaptive immunity are much stronger predictors of mortality in course of virally-suppressive cART [33–35]. Recently, biomarkers of systemic inflammation and immune activation have been demonstrated to be predictive of mortality in the first 12 months of cART in South Africa and Uganda, possibly serving as efficient prognostic indicators for patients initiating therapy [36].

Nevertheless, whether or not markers of microbial translocation and systemic inflammation pre-cART predict morbidity/mortality during suppressive cART, independently of other known risk factors, is still under debate.

Given these premises, we explored the possible associations between circulating markers of microbial translocation (LPS, sCD14, EndoCAb) and systemic inflammation (hs-CRP) measured before cART introduction and the risk of long-term disease progression upon cART start in PLWHIV enrolled in the Icona Foundation Study Cohort. Among markers of inflammation, we chose to investigate hs-CRP given its frequent routinary use in the clinical management of PLWHIV, reasoning that it might be an easy and informative biomarker to be exploited in the most personalized clinical management of cART-treated patients.

## Methods

### Study design

The study population was selected from the Icona (Italian Cohort Naïve Antiretrovirals) Foundation cohort. Icona is an Italian multicenter prospective observational cohort study, set up in 1997 and including HIV-1 infected subjects [37]. To date, the cohort has recruited more than 18,500 patients prospectively followed from 57 infectious disease wards in Italy. All patients must be antiretroviral-naïve at the time of enrolment, regardless of the reason for which they had never been treated.

Demographic, viro-immunological and clinical data as well as details on antiretroviral regimen, are registered in an electronic case report form (eCRF). All the clinical events, lab and viro-immunological parameters are registered in the electronic database from the enrollment till the last available follow-up, a follow-up visit is scheduled at least every 6 months.

Of all participants in the cohort, we selected a subset of 2376 who on January 01, 2015 had already started cART, had ≥2 vials of plasma stored over the 6 months between entry and the date of cART initiation and ≥ 1 CD4 count after cART initiation. We have then extracted a random sample of 500 patients from this universe. For 24 of these patients (5%) the second vial could not be located and were not eventually included. CD4 count and HIV-RNA are typically measured on the same collected sample, so all patients also had ≥1 viral load during cART. Death and clinical events are recorded from the information on the medical record of the patients. Death records are not linked to national or regional registry and the accuracy of AIDS-defining and non-AIDS events diagnoses are checked by both central and on-site monitoring. Central monitoring to check the accuracy of the data entered is performed every 6 months while on-site monitoring annually. Of all participants in the cohort, we selected a subset of 2376 who on January 01, 2015 had already started cART, had ≥2 vials of plasma stored over the 6 months between entry and the date of cART initiation and ≥ 1 CD4 count after cART initiation. We have then extracted a random sample of 500 patients from this universe. For 24 of these patients (5%) the second vial could not be located and were not eventually included.

The Icona Foundation study was approved by the Ethics Committee (Institutional Review Board) of each participating institution. All of the individuals enrolled provided written informed consent at the time of enrolment.

### Microbial translocation and systemic inflammation markers

On the extracted stored samples, we retrospectively measured plasma levels of microbial translocation markers, such as LPS (LAL test, Lonza Bioscience, Basel, Switzerland), sCD14 (ELISA assay, R&D Systems, a Biotechne brand, Minneapolis, MN, USA), EndoCAb (ELISA, Hycult Biotech, Uden, Netherlands) and the pro-inflammatory marker hs-C reactive protein, CRP (ELISA assay, R&D Systems, a Biotechne brand, Minneapolis, MN, USA), according to manufacturers’ instruction. Only one sample per participants stored prior tocART initiation was used.

The Limulus amoebocyte lysate (LAL) test, an assay generally used to quantify endotoxin, presents several technical issues, due to reagent contaminations, as well as to the inhibitory nature of some plasma components [38]. The protocol for LPS quantification has been optimized in our lab, in order to maximize the assay performance, adding a dilution step, followed by a heat-mediated protein denaturation step. Following this implementation, we were able to quantify plasma LPS in 278/486 (57%) samples.

### Endpoint

We defined a primary composite endpoint of clinical progression as the occurrence of AIDS-defining condition, non-AIDS severe events (SNAEs) or death whichever occurred first. SNAEs included: cardiac decompensation, chronic renal insufficiency (defined as clinical diagnosis), end-stage liver diseases, myocardial infarction, malignancies, pneumonia, end-stage renal diseases (defined as 2 consecutive measurements of eGFR< 60 ml/min at least 3 months apart) and septic infections. More details regarding the exact endpoint-defining events included are provided in Supplementary Table 1.

We next defined two alternative composite endpoints after distinguishing non-infectious related events from infectious-related events. The latter group included i) AIDS-defining infections; ii) end-stage liver diseases caused by viral agents; iii) infectious-related malignancies; iii) pneumonia; and iv) sepsis (see Supplementary Table 1 for the details). All other morbidity events were classified as non-infectious and death was counted as an event for both these alternative endpoints.

### Statistical analyses

Standard survival analysis (Kaplan-Meier curves and Cox regression models with time fixed covariate measured before cART initiation) was used to evaluate the association between MT and systemic inflammation markers and each of the defined endpoints. Participants’ follow-up accrued from the date of starting cART up to one of the endpoint defining events (only the first event was counted in the analysis). For those who did not develop any of the endpoint-defining events, was censored at time of last available clinical. Separate Cox regression models were fitted for each of the biomarkers both univariable and after controlling for key baseline time-fixed confounding factors (age, CD4, VL, HCV/HBV, years of cART, duration of HIV infection at starting cART and type of cART started). A final model was also constructed under the assumption that the association with each of the markers could have been further confounded by the remaining markers. Repeated measures of the markers were not available, and this prevented us from planning correction for potential post-baseline confounding.

MT and pro-inflammatory markers were evaluated using both a binary variable (above and below median) and as a continuous variable (in the natural logarithmic scale). Dichotomisation was based on the median value of the study population because there are no standardised clinical cut-off values for these markers. For hsCRP we have also a categorization that used the 33% percentile in order to compare the risks between patients with low and intermediate values vs. high. The 66% percentile corresponds to the clinically relevant cut-off of 5.4 mg/l. The natural logarithmic scale was used to reduce variance, increase power and comparability between the results across markers.

We also performed a number of sensitivity analyses: i) by repeating the main analysis on the subset of patients who had CD4 count less than 350 cell/mmc before cART starting, ii) after further controlling for mode of HIV transmission, smoking and alcohol consumption, iii) after excluding participants who showed hsCRP levels > 10 mg/L and iv) after restricting to those who had a complete profile for all biomarkers evaluated.

## Results

### Patients’ characteristics

We analyzed the data of 486 patients fulfilling the inclusion criteria and overall contributing to 1605 biomarker measures: 278 with LPS, 375 with hs-CRP, 477 with sCD14 and 475 with EndoCAb. Table 1 shows the main characteristics of the study population. Patients were preferentially males 347/486 (71,4%), with a median age of 37 years (range 18, 74) and a history of sex intercourses (homosexual contacts 26,3%; heterosexual contacts 47,5%). The median CD4 T-cell count was 256 cell/mmc and the median HIV viral load was 4,8 Log cp/mL .

**Table 1:** Main Characteristics of the study population

Characteristics	Total
	*N* = 486
**Gender, n (%)**	
**Female**	139 (28.6%)
**Age, years, median (range)**	37 (18, 74)
**Mode of HIV transmission, n (%)**	
**Homosexual contacts**	128 (26.3%)
**Heterosexual contacts**	232 (47.5%)
**IDU**	103 (21.2%)
**Other/unknown**	23 (4.7%)
**CD4 count, cells/mmc, median (range)**	256 (21180)
**CD4 nadir, cell/mmc, median (IQR)**	242 (121–336)
**Viral load, log10 copies/mL, median (IQR)**	4.8 (4.1, 5.2)
**Hepatitis co-infection, n (%)**	
**Negative**	176 (36.1%)
**Positive**	97 (19.9%)
**Not tested**	215 (44.1%)
**Time from HIV diagnosis to cART, months Median (IQR)**	44 (38, 50)
**Calendar year of starting cART, median (range)**	2003 (1998, 2010)
**Clinical Follow-up time**^a^**, months Median (IQR)**	79.0 (30.0, 134.0)

Note: *IQR* Interquartile range, *IDU* Injection drug users, *cART* Combination of antiretroviral therapy. ^a^Clinical follow-up time: time between cART initiation and the clinical event or for those who did not develop any of the endpoint-defining events, time between cART initiation and last CD4 count or available visit

### Effect of microbial translocation and inflammation on clinical progression

Using the primary composite endpoint of clinical progression, 125 progression events were recorded: 10.8 (0.77–14.8)/1000 PYFU new AIDS, 18.3 (14.2–23.3)/1000 PYFU SNAEs, and 5.56 (3.40–8.57)/1000 PYFU deaths, corresponding to 39 (31%) AIDS, 66 (53%) SNAEs and 20 (16%) deaths. Among the 66 SNAEs events we identified: 1 cardiac decompensation, 3 chronic renal insufficiency, 4 liver diseases, 5 myocardial infarctions, 16 malignancies, 1 myelitis, 1 pancreatitis, 11 pneumonia, 22 renal diseases, 2 septic infections. We found no major differences in the Kaplan Meier estimated cumulative probability of disease progression according to plasma LPS (log-rank *p* = 0.524), sCD14 (*p* = 0.821), EndoCAb (*p* = 0.083) and hs-CRP (*p* = 0.119) strata. Similar results were observed when we restricted the analysis to the 70 infectious-related events (data not shown).

### Identification of predictors of clinical progression

We next sought to investigate the association between pro-inflammatory and MT parameters and risk of outcomes after controlling for potentially confounding factors. Table 2 shows the results of the Cox regression analysis of time to developing the composite endpoint of clinical progression (i.e. new AIDS, SNAEs and/or death), in which biomarkers were included in the models using a binary variable (above or below the median). Interestingly, hs-CRP was the only biomarker associated with disease progression (relative hazard = 1.52 comparing hs-CRP > 1.51 vs. below 1.51, 95% CI 1.01–2.29, *p* = 0.044; Table 2). The association was a little attenuated after controlling for key confounding factors (RH = 1.47, 95% CI 0.96–2.26, *p* = 0.08) and remained virtually unchanged after further adjustment for the other biomarkers (relative hazard = 1.54 comparing > hs-CRP 1.51 vs. below 1.51, 95% CI 0.99–2.39, *p* = 0.056; Table 2).

**Table 2:** RH of clinical progression from fitting a Cox regression model

	Crude and adjusted relative hazards of new AIDS or non-AIDS Severe Events (SNAEs) or death in total population					
	Crude RH (95% CI)	***p***-value	Adjusted^a^ RH (95% CI)	***p***-value	Adjusted^b^ RH (95% CI)	***p***-value
**(*a) Biomarkers fitted as categorical variables***						
**LPS, pg/ml**						
**< =250.8**	1.0		1.0		1.0	
**> 250.8**	0.85 (0.53, 1.36)	0.495	0.76 (0.46, 1.26)	0.292	0.88 (0.52, 1.49)	0.643
**not measured**	1.09 (0.72, 1.65)	0.681	1.11 (0.72, 1.73)	0.631	1.22 (0.77, 1.92)	0.400
**sCD14, ug/ml**						
**< =2.83**	1.0		1.0		1.0	
**> 2.83**	1.10 (0.77, 1.58)	0.586	0.95 (0.65, 1.39)	0.805	0.88 (0.59, 1.29)	0.504
**not measured**	1.26 (0.40, 4.03)	0.695	0.87 (0.26, 2.86)	0.819	1.44 (0.41, 5.02)	0.570
**EndoCAb, MMU/ml**						
**< =36.5**	1.0		1.0		1.0	
**> 36.5**	0.75 (0.52, 1.06)	0.107	0.82 (0.56, 1.19)	0.296	0.84 (0.57, 1.24)	0.378
**not measured**	0.22 (0.03, 1.56)	0.129	0.19 (0.03, 1.38)	0.100	0.18 (0.02, 1.40)	0.100
**hs-CRP, mg/L**						
**< =1.51**	1.0		1.0		1.0	
**> 1.51**	1.52 (1.01, 2.29)	0.044	1.47 (0.96, 2.26)	0.077	1.54 (0.99, 2.39)	0.056
**not measured**	1.19 (0.74, 1.93)	0.478	1.26 (0.76, 2.07)	0.369	1.28 (0.77, 2.13)	0.345
***(b) Biomarkers fitted as continuous variables in the log***_***e***_ ***scale***						
**LPS, pg/ml**						
**per log**_**e**_ **higher**	0.99 (0.73, 1.35)	0.971	0.94 (0.68, 1.31)	0.729	0.72 (0.46, 1.12)	0.147
**sCD14, ug/ml**						
**per log**_**e**_ **higher**	1.17 (0.84, 1.63)	0.342	0.95 (0.64, 1.40)	0.798	0.79 (0.41, 1.51)	0.471
**EndoCAb, MMU/ml**						
**per log**_**e**_ **higher**	0.87 (0.70, 1.07)	0.195	0.94 (0.75, 1.17)	0.567	1.03 (0.70, 1.52)	0.874
**hs-CRP, mg/L**						
**per log**_**e**_ **higher**	1.12 (0.99, 1.27)	0.065	1.07 (0.94, 1.21)	0.334	1.06 (0.89, 1.25)	0.539

Note: SNAEs: cardiac decompensation, chronic renal insufficiency, liver diseases, myocardial infarction, malignancies, pneumonia, renal diseases and septic infections ^a^ All models (a separate one for each biomarker) adjusted for age, CD4, VL, HCV/HBV, years of cART, duration of HIV infection at starting cART, type of cART started ^b^Further mutually adjusted for all biomarkers

We have further investigated the association with hs-CRP by stratifying the study population according to the 33% percentiles of the distribution and creating three groups: low (0.0–1.1 mg/L), intermediate (1.2–5.3 mg/L) and high (> 5.3 mg/L). These results confirm those of the above-mentioned analysis and show a dose-response relationship with the outcome: the higher hs-CRP values, the higher the risk of clinical progression (*p* = 0.002; Table 3).

**Table 3:** RH of clinical progression from fitting a Cox regression model, according to the 33% percentiles of the distribution of hs-CRP

	Relative hazards of AIDS/SNAE/death					
hs-CRP, mg/L	Unadjusted RH (95% CI)	***p***-value	Adjusted^a^ RH (95% CI)	***p***-value	Adjusted^b^ RH (95% CI)	***p***-value
**All patients (*n*** **= 375)**						
0.0–1.1	1		1		1	**Global**
						0.002
1.2–5.3	1.42 (0.88, 2.27)	0.147	1.50 (0.89, 2.52)	0.127	1.61 (0.95, 2.73)	0.078
5.4+	1.85 (1.09, 3.15)	0.023	1.59 (0.88, 2.89)	0.128	1.69 (0.92, 3.11)	0.093
per natural log higher	1.12 (0.99, 1.27)	0.065	1.06 (0.89, 1.25)	0.539	1.12 (0.93, 1.36)	0.238
**Complete case analysis**^c^ **(*n*** **= 208)**						
0.0–1.1	1		1		1	**Global**
						0.07
1.2–5.3	1.15 (0.60, 2.20)	0.674	1.39 (0.66, 2.91)	0.386	1.70 (0.77, 3.76)	0.189
5.4+	1.43 (0.67, 3.06)	0.352	1.01 (0.41, 2.51)	0.982	1.29 (0.49, 3.42)	0.608
per natural log higher	1.11 (0.93, 1.32)	0.242	1.06 (0.89, 1.25)	0.539	1.12 (0.93, 1.36)	0.238
**Restricting to people with hs-CRP lower than 10 mg/L (*n*** **= 335)**						
0.0–1.1	1		1		1	**Global**
						0.002
1.2–5.3	1.40 (0.88, 2.25)	0.158	1.56 (0.92, 2.64)	0.098	1.78 (1.05, 3.04)	0.034
5.4+	1.28 (0.59, 2.79)	0.535	1.42 (0.57, 3.49)	0.451	1.42 (0.57, 3.56)	0.453
per natural log higher	1.02 (0.87, 1.21)	0.779	1.08 (0.84, 1.39)	0.562	1.12 (0.86, 1.47)	0.405

^a^adjusted forage, CD4, VL, HCV/HBV, year of cART, duration of HIV infection at starting cART,type of cART started as well as all other biomarkers

^b^adjusted forage, mode of HIV transmission, alcohol consumption, smoking, CD4, VL, HCV/HBV, year of cART, duration of HIV infection at starting cART,type of cART started as well as all other biomarkers

^c^complete data for all four biomarkers

Given that hsCRP levels > 10 mg/L likely represent incident infections at the time of sampling, we performed a sensitivity analysis excluding persons with levels > 10 mg/L, and we found a significant association between hs-CRP and clinical progression (*p* = 0.002; Table 3).

We have also performed a sensitivity analysis after restricting only to people with complete data for all biomarkers (278 is the number of people with LPS quantified while only 208 had a quantification for all of the markers included). Results of this analysis were similar to those of the main analysis.

We next asked whether the involvement of the pre-cART pro-inflammatory *milieu* might differently affect the pathogenesis of infectious and non-infectious related diseases in course of long-term suppressive cART. In this analysis the breakdown of the 70 infectious-related events were as follows: 39 AIDS-defining infections, 5 infectious-related malignancies, 4 viral hepatitis, 2 septic infections, 11 pneumonia and 9 infectious-driven death. Although the association was weaker, possibly because of the reduced statistical power, again, hs-CRP was the biomarkers which was most strongly associated with clinical progression after controlling for confounders and for the other biomarkers (relative hazard = 1.71 comparing hs-CRP > 1.51 vs. below 1,51, 95% CI 0.94–3.13, *p* = .081; Table 4.part A). On the opposite, EndoCAb was associated with non-infectious related events, when the biomarker was fitted as categorical variable (relative hazard = 0.52 comparing EndoCAb > 36.5 vs. below 36.5, 95% CI 0.29, 0.92, *p* = .026), as well as when it was fitted as continuous variable (relative hazard = 0.68, 95% CI 0.48, 0.97, *p* = .031). However, the association was lost after controlling for confounders and for the other biomarkers (Table 4.part B).

**Table 4:** RH of clinical progression, defined as the occurrence of an infectious-related or non-infectious related event, from fitting a Cox regression model

**PART A. infectious related events**	**Crude and adjusted relative hazards of infectious-related events in total population**					
	**Crude RH** **(95% CI)**	***p*-value**	Adjusted^*^ **RH** **(95% CI)**	***p*-value**	Adjusted^**^ **RH** **(95% CI)**	***p*-value**
**(*a) Biomarkers fitted as categorical variables***						
**LPS, pg/ml**						
**< =250.8**	1.0		1.0		1.0	
**> 250.8**	0.79 (0.40, 1.56)	0.506	0.67 (0.33, 1.35)	0.262	0.75 (0.36, 1.54)	0.430
**not measured**	1.34 (0.77, 2.33)	0.305	1.14 (0.63, 2.06)	0.658	1.19 (0.65, 2.19)	0.567
**sCD14, ug/ml**						
**< =2.83**	1.0		1.0		1.0	
**> 2.83**	1.45 (0.89, 2.35)	0.134	1.19 (0.71, 1.99)	0.511	1.08 (0.64, 1.82)	0.787
**not measured**	1.83 (0.43, 7.67)	0.411	1.08 (0.25, 4.73)	0.918	1.71 (0.35, 8.37)	0.508
**EndoCAb, MMU/ml**						
**< =36.5**	1.0		1.0		1.0	
**> 36.5**	0.94 (0.59, 1.51)	0.811	0.95 (0.58, 1.55)	0.826	0.98 (0.59, 1.63)	0.942
**not measured**	0.48 (0.07, 3.50)	0.469	0.34 (0.05, 2.52)	0.290	0.35 (0.04, 3.13)	0.351
**hs-CRP, mg/L**						
**< =1.51**	1.0		1.0		1.0	
**> 1.51**	1.58 (0.91, 2.74)	0.103	1.64 (0.92, 2.95)	0.096	1.71 (0.94, 3.13)	0.081
**not measured**	1.31 (0.69, 2.49)	0.407	1.47 (0.75, 2.87)	0.260	1.49 (0.75, 2.96)	0.250
***(b) Biomarkers fitted as continuous variables in the log***_***e***_ ***scale***						
**LPS, pg/ml**						
**per log**_**e**_ **higher**	0.96 (0.62, 1.49)	0.857	0.85 (0.54, 1.34)	0.486	0.58 (0.31, 1.09)	0.089
**sCD14, ug/ml**						
**per log**_**e**_ **higher**	1.21 (0.82, 1.80)	0.344	0.92 (0.57, 1.50)	0.747	1.01 (0.54, 1.90)	0.968
**EndoCAb, MMU/ml**						
**per log**_**e**_ **higher**	1.00 (0.76, 1.32)	0.982	1.01 (0.76, 1.35)	0.927	0.95 (0.56, 1.61)	0.857
**hs-CRP, mg/L**						
**per log**_**e**_ **higher**	1.09 (0.92, 1.28)	0.316	1.05 (0.89, 1.24)	0.556	1.13 (0.89, 1.43)	0.303
**PART B. Non-infectious related events**	**Crude and adjusted relative hazards of non-infectious related events in total population**					
	**Crude RH** **(95% CI)**	***p*-value**	**Adjusted**^a^ **RH** **(95% CI)**	***p*-value**	**Adjusted**^b^ **RH** **(95% CI)**	***p*-value**
**(*a) Biomarkers fitted as categorical variables***						
**LPS, pg/ml**						
**< =250.8**	1.0		1.0		1.0	
**> 250.8**	0.84 (0.42, 1.67)	0.625	0.88 (0.42, 1.85)	0.728	1.08 (0.49, 2.37)	0.856
**not measured**	0.72 (0.38, 1.39)	0.327	0.95 (0.47, 1.92)	0.892	1.12 (0.54, 2.34)	0.758
**sCD14, ug/ml**						
**< =2.83**	1.0		1.0		1.0	
**> 2.83**	0.71 (0.41, 1.25)	0.241	0.69 (0.38, 1.27)	0.232	0.63 (0.34, 1.17)	0.147
**not measured**	0.78 (0.11, 5.72)	0.805	0.68 (0.09, 5.21)	0.712	1.16 (0.14, 9.60)	0.889
**EndoCAb, MMU/ml**						
**< =36.5**	1.0		1.0		1.0	
**> 36.5**	0.52 (0.29, 0.92)	0.026	0.64 (0.35, 1.19)	0.163	0.64 (0.33, 1.22)	0.172
**not measured**	0.00 (0.00,.)	0.986	0.00 (0.00,.)	0.987	0.00 (0.00,.)	0.986
**hs-CRP, mg/L**						
**< =1.51**	1.0		1.0		1.0	
**> 1.51**	1.41 (0.75, 2.65)	0.281	1.25 (0.65, 2.43)	0.505	1.30 (0.65, 2.58)	0.454
**not measured**	1.04 (0.49, 2.22)	0.921	0.99 (0.46, 2.16)	0.985	0.96 (0.42, 2.16)	0.917
***(b) Biomarkers fitted as continuous variables in the log***_***e***_ ***scale***						
**LPS, pg/ml**						
**per log**_**e**_ **higher**	0.87 (0.56, 1.36)	0.542	0.87 (0.52, 1.44)	0.592	0.89 (0.46, 1.71)	0.730
**sCD14, ug/ml**						
**per log**_**e**_ **higher**	1.02 (0.56, 1.86)	0.943	0.95 (0.49, 1.87)	0.887	0.52 (0.18, 1.50)	0.228
**EndoCAb, MMU/ml**						
**per log**_**e**_ **higher**	0.68 (0.48, 0.97)	0.031	0.81 (0.56, 1.16)	0.243	1.07 (0.59, 1.91)	0.830
**hs-CRP, mg/L**						
**per log**_**e**_ **higher**	1.14 (0.94, 1.38)	0.196	1.05 (0.85, 1.30)	0.643	0.96 (0.73, 1.26)	0.750

Note: ^a^ All models (a separate one for each biomarker) adjusted for age, CD4, VL, HCV/HBV, years of cART, duration of HIV infection at starting cART, type of cART started ^b^Further mutually adjusted for all biomarkers

The sensitive analysis restricted to PLWHIV in Icona who started cART with CD4 count < 350/mmc showed no association between hs-CRP and the two clinical composite endpoints (data not shown).

## Discussion

Over years of successful cART, serious non-AIDS events (SNAEs) have become the major causes of morbidity and mortality in PLWHIV [39]. Despite the complexity of SNAEs pathogenesis, persistent immune activation and inflammation appear important contributors in PLWHIV on effective treatment [7, 40]. Besides, patients with incomplete CD4 recovery have been shown higher residual immune activation/inflammation, also possibly linked to microbial translocation [14, 15, 27, 28].

Here we have evaluated the association between pre-therapy systemic inflammation and microbial translocation and clinical outcome measured after cART.

Our data show some evidence for an association between pre-therapy circulating hs-CRP, but not microbial translocation, and higher risk of clinical progression after cART initiation, regardless of patients’ key confounding factors such as age and nadir CD4 count, in all suggesting that pre-therapy HIV-driven pro-inflammatory *milieu* might be a more important mediator of long-term HIV-disease progression.

While both IL-6 and D-dimer have been shown to be independently associated with serious non-AIDS conditions or death among HIV-positive adults with suppressed virus [41], posing them as ideal biomarkers for interventional strategies, hs-CRP involvement as correlate of disease progression is less clear. Despite its lack of specificity, hs-CRP (an acute phase protein produced by the liver following general inflammation) has significant predictive value in different medical conditions [42–44], and is often measured during routine laboratory exams in PLWHIV. We therefore reasoned that a better understanding of the possible associations between hs-CRP levels and clinical outcomes in cART-treated patients might provide an additional value for the treating physician: should such an association be found relevant, hs-CRP monitoring during follow-up visits might indeed help clinicians in paving tailored management strategies for their patients. Our data carries evidence that levels of pre-therapy hs-CRP are associated with greater risk of clinical progression and support its possible clinical role value in the management of PLWHIV.

Interestingly, by performing the same analysis according to infectious vs non-infectious clinical conditions, a similar yet non-significant association was retained uniquely between hs-CRP and infectious-related events, whereas a much weaker association was shown between EndoCAb and non-infectious diseases. Given that inflammation, as well as endotoxin immunity, has been shown to have a pathogenetic role in both infectious and non-infectious diseases, further studies should specifically address this issue in well-defined cohorts of HIV+ patients on cART.

MT and activation/inflammation markers have been associated with mortality and disease progression among cART initiators in some, but not all studies [23, 30, 45, 46]. This discrepancy might be due to the temporality and the setting in which biomarkers were measured (some in untreated patients and others in PLWHIV receiving cART).

According to a recent hypothesis [40], the root drivers of inflammation reflected by systemic biomarker measurements and the disease manifestations induced by inflammation are likely to differ according to nadir CD4. Indeed, several factors have been called upon as potential contributors of ongoing inflammation during suppressive cART, and include residual low-level viral replication, co-infections, gut damage with continuous passage of microbial byproducts in the systemic circulation.

In our analysis, circulating MT markers failed to predict disease progression even after restricting the analyses to PLWHIV with severe pre-cART CD4 below 350 cell/mmc, suggesting that other factors probably encompass the effect of MT alone in contributing to disease progression. Whilst seemingly divergent from data by Hunt et al. [29] that demonstrate an association between on-therapy markers of gut damage/MT and mortality, our findings support the hypothesis that the clinical predictive role of gut-damage measured before cART might be outweighed by pre-therapy inflammation and that it might assume greater relevance later on in the setting of effective therapy.

Several limitations to our study need to be acknowledged. First, CD4 count nadir of our study population was low (243 cells/mm3 on average) and this could have influenced the results of the analysis. Further data are needed to confirm the role of both pre-therapy inflammation and MT as clinical predictors also in individuals who initiate cART earlier in the infection. Secondly, our results rely on the usual assumption of a correctly specified model (e.g. all measured confounders have been correctly accounted for) and no unmeasured confounding. We cannot rule out that the lack of association is masked by residual or unmeasured confounding factors. In a sensitivity analysis we further controlled for mode of HIV transmission, alcohol consumption and smoking and none of these adjustments attenuated the association to the null. Third, selection bias could be an issue due to the non-negligible proportion of participants with missing data for one or more of the biomarkers (namely LPS was only available for 278 and hsCRP for 375 participants). However, main characteristics of participants with missing values and risk of outcomes were similar to those of the other analyzed groups suggesting that non-successful testing had occurred at random. Importantly, although all of the tested hypotheses have been pre-specified, many comparisons were performed without controlling for possible inflation of the type I error. Finally, the high quality of care received by patients enrolled in our study could have led to a lower number of clinical events, compared to other studies, possibly limiting the power of the analysis.

While our study failed to find an association between LPS and disease progression in our cohort of PLWHIV, it must be acknowledged that LPS measurement might not entirely capture the extent of MT, given its fluctuation during the day and its association with food intake and the fact that repeated measures of the markers post cART initiation were not available. A deeper understanding of the role of MT as cause of inflammation and disease progression in the course of cART could be gained by the comprehensive assessment of additional and reliable markers of both MT and inflammation, including the fungal translocation marker Beta d glucan (BDG) that has proven to maintaining stable plasma levels over 24 h, with stronger association with pro-inflammatory markers [47–49]. Also, a most comprehensive assessment of the interaction between several markers of microbial translocation and inflammation including IL-6 and D-dimer levels is strongly advocated to most finely unravel the possible mechanistic links between inflammation, MT and disease progression upon virally-suppressive cART.

## Conclusions

Our analysis suggests that the inflammatory marker hs-CRP measured before cART initiation is a predictor of long-term clinical disease progression. As a consequence, more frequent clinical monitoring and disease-specific screening after cART initiation should be considered for PLWHIV showing high levels of hs-CRP while still untreated.

To date, a number of studies have been conducted to evaluate strategies aimed to reduce persistent immune activation on ART, such as cART intensification, treatment of co-infections, the use of anti-inflammatory agents and improvement of immune recovery through cytokine administration (as reviewed in 7 [7]), but there is little conclusive evidence on which strategy will result in clinical benefits. Further research efforts are needed to improve our current understanding of the pathways that connect HIV virus, immune activation/inflammation and the cascade of events leading to the SNAEs before firmly indicating hs-CRP as the main candidate to guide our clinical decisions.

## Supplementary Information

**Additional file 1.**

## Data Availability

The datasets used and/or analyzed during this study are available from the corresponding author on reasonable request.

## Abbreviations

cART: Combined antiretroviral treatment
HIV: Human immunodeficiency virus
PLWHIV: People living with HIV
MT: Microbial translocation
LPS: Lipopolysaccharide
sCD14: Soluble CD14
EndoCAb: Endotoxin core antibodies
hs-CRP: High sensitive C reactive protein
SNAEs: Serious non-AIDS events
PYFU: Per year follow-up
eGFR: Estimated Glomerular Filtration Rate
VL: Viral load
HCV: Hepatitis C virus
HBV: Hepatitis B virus
IQR: Interquartile range.
