# Correction to: Inflammation and microbial translocation measured prior to combination antiretroviral therapy (cART) and long-term probability of clinical progression in people living with HIV

**DOI:** 10.1186/s12879-021-06330-1

**Published:** 2021-06-24

**Authors:** Esther Merlini, Alessandro Cozzi-lepri, Antonella Castagna, Andrea Costantini, Sergio Lo Caputo, Stefania Carrara, Eugenia Quiros-Roldan, Maria A. Ursitti, Andrea Antinori, Antonella D’Arminio Monforte, Giulia Marchetti

**Affiliations:** 1 Clinic of Infectious Diseases, Department of Health Sciences, University of Milan, “ASST Santi Paolo e Carlo, Milan, Italy; 2 Institute for Global Health, University College London, London, UK; 3 Department of Infectious Diseases, IRCCS San Raffaele Scientific Institute, University Vita-Salute San Raffael, Milan, Italy; 4 Clinical Immunology Unit, Azienda Ospedaliero-Universitaria Ospedali Riuniti, Marche Polytechnic University, Ancona, Italy; 5 Infectious Diseases Unit, University of Foggia, Foggia, Italy; 6 Microbiology Biobank and Cell Factory Unit, National Institute for Infectious Diseases ‘Lazzaro Spallanzani’ IRCCS, Rome, Italy; 7 University Department of Infectious and Tropical Diseases, University of Brescia and ASST Spedali Civili, Brescia, Italy; 8 Department of Infectious Diseases, S. Maria Nuova IRCCS Hospital, Reggio Emilia, Italy; 9 HIV/ AIDS Department, INMI, L. Spallanzani, IRCCS, Rome, Italy

**Correction to: BMC Infect Dis 21, 557 (2021)**

**https://doi.org/10.1186/s12879-021-06260-y**

Following publication of the original article [1], the authors identified an error in the author’s name of Eugenia Quiros Roldan.

The incorrect name is:

Given name: Eugenia Quiros

Family name: Roldan

The correct name is

Given name: Eugenia

Family name: Quiros-Roldan

The original article has been corrected as well.
