# Supplementary material for: Inflammation and microbial translocation measured prior to combination antiretroviral therapy (cART) and long-term probability of clinical progression in people living with HIV
Source: BMC Infect Dis. 2021 Jun 12;21:557. doi: 10.1186/s12879-021-06260-y (PMC8196504; doi:10.1186/s12879-021-06260-y)
Supplement: Supplementary file 1 — Additional file 1. [file 12879_2021_6260_MOESM1_ESM.docx]

**Supplementary Table 1. Infectious and non-infectious clinical events**

| **Event** | **N= 125** | **%** |
| --- | --- | --- |
| **AIDS** | **39** | **31,20** |
| PCP | 5 | 12,80 |
| Malignant Lymphoma | 5 | 12,80 |
| Esophageal Candidiasis | 5 | 12,80 |
| Kaposi’s Sarcoma | 4 | 10,20 |
| Tubercolosis | 4 | 10,20 |
| Non-tubercolar mycobacteriosis | 3 | 7,70 |
| PML | 2 | 5,10 |
| Bacterial Pneumonia (>=2 events/year) | 2 | 5,10 |
| Extra-pulmonary Cryptococcosis | 2 | 5,10 |
| Wasting syndrome | 1 | 2,60 |
| AIDS dementia complex | 1 | 2,60 |
| Cervix Cancer | 1 | 2,60 |
| Cerebral Toxoplasmosis | 1 | 2,60 |
| Herpes Simplex – ulcers (>1 months) | 1 | 2,60 |
| Visceral Leishmaniosis | 1 | 2,60 |
| CMV | 1 | 2,60 |
| **Cardiac decompensation** | 1 | 0,80 |
| **Death** | 20 | 16,00 |
| AIDS-related | 7 | 35.00 |
| HCV-related | 2 | 10.00 |
| **IRC** | 3 | 2,40 |
| **Liver disease, HCV-related** | 4 | 3,20 |
| **MI** | 5 | 4,00 |
| **Malignancies** | 16 | 12,80 |
| HPV-related | 3 | 17,80 |
| HCV-related | 2 | 12,50 |
| **Meningitis** | 1 | 0,80 |
| **Pancreatitis** | 1 | 0,80 |
| **Pneumonia** | 11 | 8,80 |
| **Renal disease** | 22 | 17,60 |
| **Septic infection** | 2 | 1,60 |

Note: PCP: P. Carini Pneumonia; PML: progressive multifocal leukoencephalopathy; AIDS: Acquired Immuno-Deficiency Sindrome; CMV: cytomegalovirus; HCV: hepatitis C virus; HPV: human papillomavirus
